# Mountain Ultra-Marathon (UTMB) Impact on Usual and Emerging Cardiac Biomarkers

**DOI:** 10.3389/fcvm.2022.856223

**Published:** 2022-03-24

**Authors:** Caroline Le Goff, Laurent Gergelé, Laurence Seidel, Etienne Cavalier, Jean-François Kaux

**Affiliations:** ^1^Department of Clinical Chemistry, University Hospital of Liege and University of Liège, Liège, Belgium; ^2^Department of Intensive Care, Intensive Unit Care, University Hospital of Saint Etienne, University of Lyon, Lyon, France; ^3^Biostatistics Department, University Hospital of Liege, Liège, Belgium; ^4^Department of Sports and Rehabilitation Sciences, University of Liège, Liège, Belgium; ^5^Physical Medicine and Sport Traumatology Department, SportS2, IOC Research Centre for Prevention of Injury and Protection of Athlete Health FIFA Medical Centre of Excellence, FIMS Collaborative Centre of Sports Medicine, University Hospital of Liège, Liège, Belgium

**Keywords:** cardiac biomarker, ultra-trail, copeptin, troponin, H-FABP, ST2

## Abstract

The number of participants in ultra-marathons is increasing. However, the data regarding the impact of this type of exercise on the cardiovascular system are contradictory. In our study, 28 ultra-trail runners were enrolled. Blood samples were collected at three time points: immediately before, immediately after, and 7 days after the ultra-marathon. Different biomarkers were measured. Immediately after the race, the blood concentrations of the different cardiac and inflammatory biomarkers increased significantly. Interestingly, some biomarkers remained high even after 7 days of recovery.

## Introduction

Endurance exercise has been demonstrated to be beneficial for cardiovascular pathologies, which have been considered the leading cause of death worldwide for the last 15 years (1). The type of endurance exercise investigated in this study was an ultra-marathon, which is defined as a foot race with a length of more than 42.2 km (2) or 50 km (3). Some of these races may be completed over multiple days, with the champions having covered the most distance in a set time period, or the races cover a specific distance, with the champions having completed the set distance in the shortest time (2). During these events, the physiological capacity of humans to adapt to extreme physical and mental conditions is challenged. Ultra-endurance races are a particularly interesting model for investigating the limits of the human body's adaptive reaction (2). One of the reasons why it is important to understand the process of this type of exercise is that mountain ultra-marathons have become very popular over the past decade (2).

**Figure 1:**
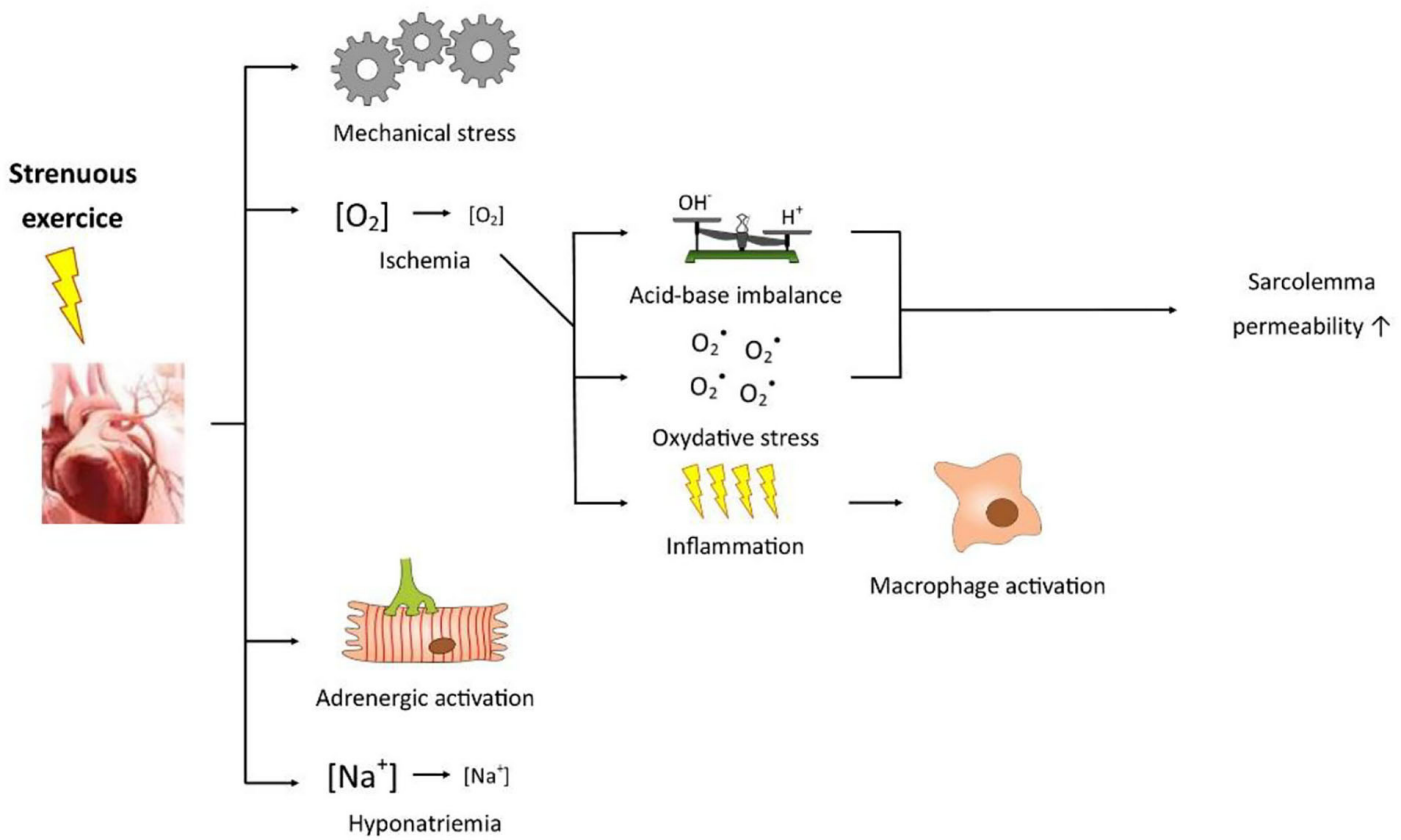
Figure showing how a prolonged strenuous exercise may impact normal cardiovascular physiological processes.

Prolonged strenuous exercise can alter normal physiological processes and can induce severe muscle damage, even in the heart; an imbalance between fluid and electrolyte levels; changes in immune function; and increased inflammation (Figure 1) (4).

The exact underlying mechanism by which these biomarkers increase, reflecting physiological or even pathophysiological changes, is unknown, and compared to well-trained runners, those who are not well-trained might exhibit a higher cardiac risk.

The aim of the study was to assess the changes in various cardiac biomarkers, including emerging remodeling and fibrosis biomarkers, after a mountain ultra-marathon measuring 105 km.

## Materials and Methods

After the local ethical committee approved the study, 28 volunteer runners, 17 of whom were male, participating in the Ultra-Trail du Mont-Blanc (105 km, total elevation gain: 5600 m) were enrolled in our study (Table 1). None of the participants were smokers, had a history of diseases, including cardiac disease symptoms in particular, and must present a certificate of no contraindication to the practice of the sport.

**Table 1:** Baseline characteristics of the volunteer runners.

**Gender**	**Age**	**Height**	**Weight**	**BMI**
Female (*n* = 11)	45,3 ± 7,8	164 ± 3,8	56,8 ± 5,4	21,1 ± 1,8
Male (*n* = 17)	43,3 ± 9,8	176,7 ± 6,1	73,6 ± 7,6	23,5 ± 2,0

*Results reported as means ± SD*.

Blood samples were collected at 3 different times: before the race (Pre), within 1 h after the race (Post) and at 7 days after the race (D + 7). Several biomarkers of heart disease [muscle and brain (MB) isoforms of creatine kinase (CK-MB)], high-sensitivity troponin T (hsTnT), copeptin and heart-fatty acid binding protein (H-FABP), of heart failure [N-terminal natriuretic peptide (NT-proBNP)], of cardiac fibrosis and remodeling [suppression of tumorigenicity 2 (ST2)] and of inflammation [high-sensitivity C-reactive protein (hsCRP)] were assayed with different analysers, such as COBAS^®^ (for CK-MB, hsTnT, NT-proBNP, H-FABP and hsCRP) and KRYPTOR^®^ (for copeptin). ST2 was measured manually with the Presage kit from CRITICAL DIAGNOSTIC^®^.

The data are presented as the mean ± SD and are stratified by the sampling time for biomarkers. Some parameters were log-transformed for normalization.

For each biomarker, the change between two time points (pre- and mid-, for example) was defined as the difference between the values measured at these time points. The change values were tested by Student's paired *t*-test. The results were considered significant at the 5% critical level (*p* < 0.05). Data analysis was carried out with SAS (version 9.4 for Windows).

## Results

**Figure 2:**
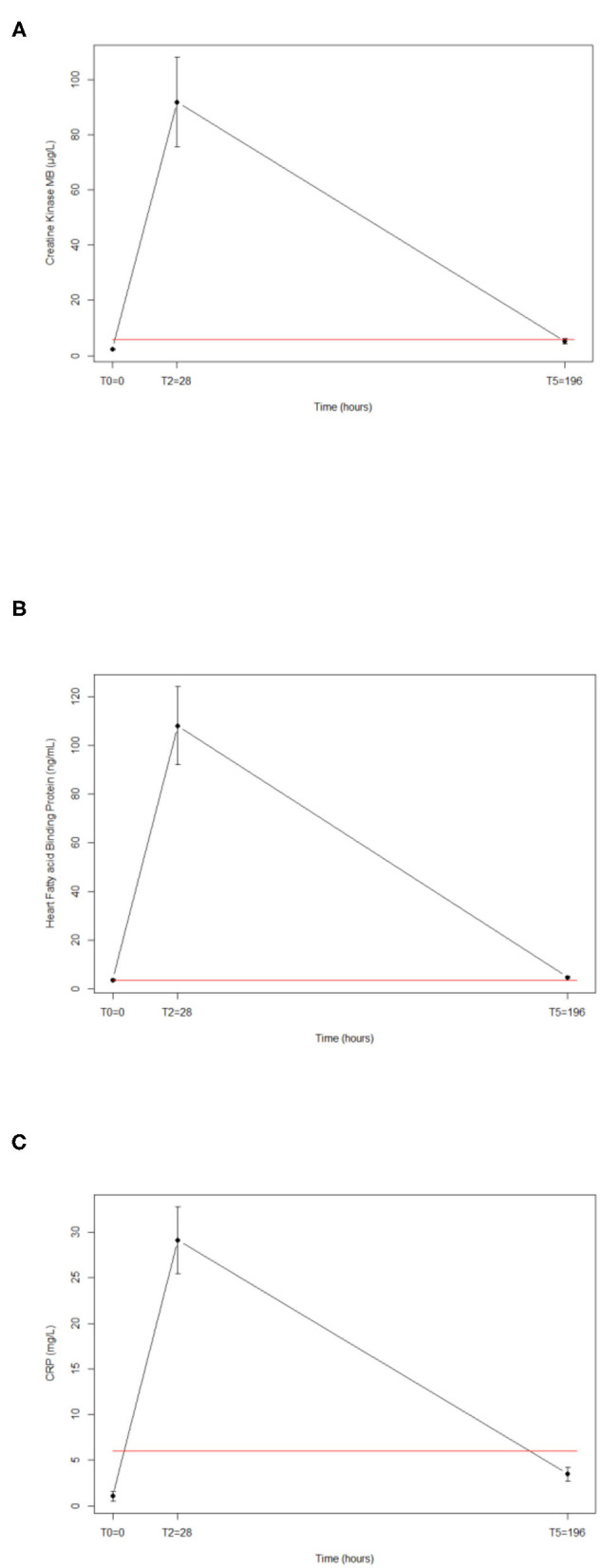
Release of cardiac biomarkers induced by a 105-km (5600 m of total elevation gain) run for CKMB **(A)**, HFABP **(C)**, and hsCRP **(B)**. Red line represents the cut-off value.

All the results are summarized in the Table 2. The serum levels of the cardiac markers [CK-MB (Figure 2A), hsTnT, NT-proBNP, copeptin, H-FABP (Figure 2B), ST2] and inflammation marker [hsCRP (Figure 2C)] increased significantly at Post. The mean values increased from Pre to Post as follows: 2.3 to 91.9 UI/L for CK-MB (*p* < 0.0001); 7.6 to 31.7 μg/L for hsTnT (*p* < 0.0001); 41.7 to 1190.5 ng/L for NT-proBNP, 4.2 to 22.9 pmol/L for copeptin (*p* < 0.0001); 3.6 to 107.8 ng/mL for H-FABP (*p* < 0.0001), 29.7 to 126.2 ng/mL for ST2 (*p* < 0.0001); and 1.05 to 29.1 mg/L for hsCRP (*p* < 0.0001). With the exception of a few (CK-MB, H-FABP, hsCRP) biomarkers in some subjects, all the values returned to the Pre-values at D + 7 (Figures 2A–C respectively).

**Table 2:** Release of cardiac biomarkers induced by a 105-km (5600 m of total elevation gain) run.

**Biomarker**	**Pre**	**Post**	**D + 7**	***P*-value**	***P*-value**
				**Pre vs.** **Post**	**Pre vs.** **D + 7**
CK-MB (μg/L)	2.31 ± 1.25	91.9 ± 86.2	5.15 ± 2.81	<0.0001	0.0002
hsTnT (ng/L)	7.60 ± 7.78	31.7 ± 22.6	7.42 ± 2.01	<0.0001	0.71
NT-proBNP (ng/L)	41.7 ± 30.5	1190 ± 663	29.5 ± 20.3	<0.0001	0.14
Copeptin (pmol/L)	4.23 ± 2.54	22.9 ± 11.5	5.82 ± 2.74	<0.0001	0.45
H-FABP (ng/mL)	3.63 ± 1.93	108 ± 85.1	4.74 ± 1.54	<0.0001	0.0052
ST2 (ng/mL)	29.7 ± 10.6	126 ± 46.6	35.5 ± 7.78	<0.0001	0.15
hsCRP (mg/L)	1.05 ± 2.95	29.1 ± 19.5	3.47 ± 2.50	<0.0001	<0.0001

*Results reported as means ± SD*.

## Discussion

Long durations of strenuous running exercise have been shown to increase cardiac biomarkers, and the mechanism by which they increase is suspected to relate to myocardial processes. This idea has been further supported by data demonstrating there are strong associations between elevated cardiac biomarkers and the post-race development of abnormalities observed in 2-dimensional echocardiograms (5, 6). Acute physiologic changes in ultra-marathoners have been observed in many different studies. Many of these studies show short-term fluctuations in biomarkers. However, it is not clear whether these changes are adaptive or pathological, and the long-term effects of these changes are not well-understood (7). Increased CK-MB levels do not definitively indicate cardiac damage, as runners have increased CK-MB levels originating from skeletal muscles as well (8).

Cardiac troponin T is currently regarded as the gold standard reference marker for myocardial necrosis due to its excellent sensitivity and cardiospecificity (9). Therefore, this biomarker increases when myocyte injury occurs. In addition, H-FABP can be a sign of ischaemia, as defined by the European Society of Cardiology and the American Heart Association (10). Previous studies have suggested that exercise-induced hsTnT increases can correspond to a benign reversible physiologic phenomenon, but the indications of this parameter are controversial. Indeed, the question of the clinical implication of this increase in troponins is inevitable. Several studies have already shown that the kinetics of the increase are similar to those observed during a cardiac event. However, the peak is always lower and the recovery is faster (11, 12).

In the era of Covid-19, it is important to consider the risk stratification of cardiac events in athletes. Indeed, during a Covid-19 infection, one could develop myocarditis. After this, athletes should ideally stop competing for 3 to 6 months because of the increased risk of cardiac events during this period. However, it is not easy to stop competing because of the risk of disqualification from other events, etc. Thus, it is important to have tools available to try to stratify the cardiac risk in these athletes. Thus, biomarkers such as ultra-sensitive troponin but also cardiac magnetic resonance imaging could help to highlight the risk of cardiac events (13–15).

Various phenomena, such as myocyte stretching, causing an increase in pressure or volume, and neurohormonal activation can explain the increases in copeptin and NT-proBNP (16). ST2 is a biomarker of cardiac remodeling and fibrosis (17, 18). ST2 is considered a very promising biomarker of heart failure and hasbeen proposed to be the new gold standard biomarker for heart failure prognostication (19). An increase in the ST2 concentration in blood can be caused by an acute increase in cardiac load (20). However, it is also a marker of remodeling and cardiac fibrosis, and it can indicate the development of arrhythmia following myocardial fibrosis. However, the ST2 concentration tends to return to normal values after 7 days of recovery, which is favorable. However, because the level at D + 7 was higher than that at Pre, we think that there is an accumulation of the effect of repeated exercise, with possible long-term consequences.

CRP is an acute-phase compound that tends to increase following a strenuous and long bout of exercise and/or muscular injury, and we observed this change after UTMB, with the maximum increase occurring at Post. Other studies on long-distance running have already shown that strenuous exercise induces muscle damage and a non-specific inflammatory response (evidenced by an increase in CRP concentration) (21–23).

## Limitations

The main limitation of our study is the small number of subjects but it is particularly difficult to recruit more subjects in this type of race and also long after the race has finished. Regarding the follow-up, we took blood samples before, at the end and 7 days after the end of the race. Of course, it might be interesting to have a longer view, but our follow-up was already longer than that done in other studies on similar events. However, this 7-day follow-up can only give an idea of the acute effects of this intense exercise. The final limitation of this study is that we were unable to perform cardiac MRI to correlate the results of our various cardiac markers with imaging, which would allow us to confirm or refute our theories in view of the observed increases.

## Conclusions

Because biomarker values tended to return within the normal reference range values within 7 days after the race, our study suggests that no permanent structural damage to the myocardium is occurring. On average, we see that almost all of them are higher than the baseline values after 7 days recovery. Statistically, we observed that only the concentrations of CKMB, HFABP, and hsCRP had not returned to baseline. However, the half-life of the biomarker must always be taken into account. This obviously plays a role in the rate of elimination of the biomarkers. Thus, if we had taken a sample even later, it is possible that all biomarkers would have returned to baseline values.

That noted, it is also possible that the effects of running accumulate over time. These observations need to be confirmed by an MRI study correlated with cardiac biomarkers of interest, such as ST2, which is a good biomarker of the development of cardiac remodeling and fibrosis.

## Data Availability Statement

The original contributions presented in the study are included in the article/supplementary material, further inquiries can be directed to the corresponding authors.

## Ethics Statement

The studies involving human participants were reviewed and approved by Comité d'éthique Hospitalo-Facultaire Université de Liège. The patients/participants provided their written informed consent to participate in this study.

## Author Contributions

CL, LG, EC, and J-FK contributed to the conception and design of the study. CL organized the database and wrote the first draft of the manuscript. CL and LS performed the statistical analysis. All authors contributed to manuscript revision, read and approved the submitted version.

## Conflict of Interest

The authors declare that the research was conducted in the absence of any commercial or financial relationships that could be construed as a potential conflict of interest.

## Publisher's Note

All claims expressed in this article are solely those of the authors and do not necessarily represent those of their affiliated organizations, or those of the publisher, the editors and the reviewers. Any product that may be evaluated in this article, or claim that may be made by its manufacturer, is not guaranteed or endorsed by the publisher.
